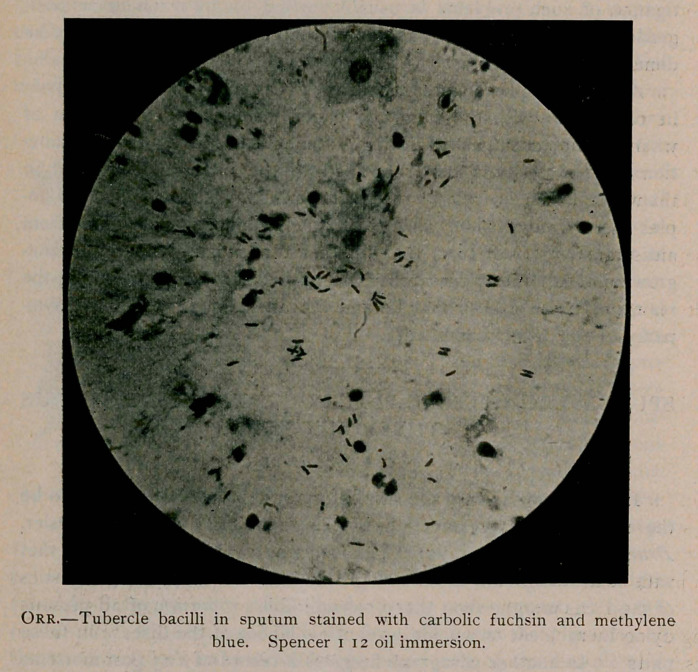# Sputum from Public Places Containing Bacillus Tuberculosis

**Published:** 1898-05

**Authors:** C. R. Orr

**Affiliations:** Of the Pathological Laboratory of the University of Buffalo


### SPUTUM FROM PUBLIC PLACES CONTAINING BACILLUS TUBERCULOSIS.

By C. R. ORR, M. D.,
Of the Pathological Laboratory of the University of Buffalo,

WITH AN INTRODUCTION

### ON LABORATORY METHODS IN MEDICINE.[1]

By HERBERT U. WILLIAMS, M. D.,
Professor of pathology, University of Buffalo.

INTRODUCTION BY DR. WILLIAMS.

THE death of Dr. Edward C. Seguin, of New York, recently, who was prominent in introducing the clinical thermometer in America, reminds us that it is only about twenty-five years since that instrument has come into general use in medicine. The thermometer was one of the first of a series of devices destined to place the diagnosis of disease upon a footing with other exact sciences. These instruments, unfortunately, are seldom so compact and simple of application as the thermometer and can generally be employed only in the office or laboratory. So a whole series of manipulations has arisen—like the counting of the number of blood corpuscles in a given volume of blood, the detection of tubercle bacilli—which make laboratory methods a necessary part of the physician's equipment. Every doctor, nowadays, must either carry on this work himself or secure some one to do it for him. Each year sees the tendency toward the employment of

Broncho-pneumonia.

1. From the University of Buffalo Annual.

exact methods becoming more pronounced. Intelligent patients are beginning to expect to see them used, and to ask for them. The medical schools, and it may be said to their credit, have not only been equal to the situation in teaching their students these new procedures, but they have anticipated the demand. Most of the exact methods have originated from purely scientific studies, carried on by men connected with some of the schools.

Lack of space will not permit any account of the functions of laboratories for chemistry, physiology and therapeutics. It is particularly to the branches in which the microscope becomes one of the indispensable tools of the student that I wish to call attention. For it is along those lines that the most radical changes have taken place in recent years. They are (1) the study of the microscopic structure of the parts of the normal body (normal histology); (2) the study of the same parts when diseased (pathological histology); (3) the action of bacteria in producing disease (bacteriology); (4) the application of exact methods to the detection of disease, which I mentioned in commencing.

Epithelioma, infiltrating the wall of the esophagus.

It is plain that the information derived from such work must be an essential part of a doctor's education. But scarcely less valuable is the training which leads to the acquirement of habits of delicacy and precision. It is my firm belief that the physician of the future will be a more careful man, as a result of the discipline of the laboratory, and that surgeons and specialists will be more painstaking and methodical. The teaching in these laboratories brings the students actually in contact with the matter to be studied more closely than in almost any other department. To ensure the careful examination of each preparation the student makes a drawing of it, which not only compels accurate observation on his part, but enables the instructor to determine whether or not it has been rightly seen and understood. Two samples of such drawings, which are average specimens, will show how well one making no claim to artistic ability can interpret what is before him.

The laboratories of our schools and hospitals have another duty to perform, no less important than that of teaching beginners. It is the study of the great problems which have as yet defied solution. For, notwithstanding the progress that has been made in this nineteenth century, we can only say that we have begun to know about a few things and many things we do not understand at all. The investigation of such problems is usually carried on by teachers or postgraduates, but our school has found that in a small way it may be done by undergraduates.

A sample of photomicrographic work (Orr tubercle bacilli) is given in connection with the following paper, which is an illustration of what can be accomplished during a student's leisure hours. No function of a university is better entitled to the interest of the public than this, which is called "research work." When, to select examples merely, we consider what great good to humanity has come from antiseptic surgery or from the diphtheria antitoxin, both purely outgrowths of laboratory researches, we appreciate how far-reaching the results of those studies may be and we can claim for them the sympathy of the whole community.

---

### SPUTUM FROM PUBLIC PLACES CONTAINING BACILLUS TUBERCULOSIS.

By C. R. ORR, M. D.

In tubercular disease the respiratory system has been shown to be the seat of primary infection in a large majority of cases. Osler, *Practice of Medicine*, says: "The frequency with which foci are met with in the lungs and bronchial glands is extraordinary and statistics of the Paris morgue show that a considerable proportion of all persons dying by accident or suicide present evidences of the disease in these parts." In another paragraph he gives a record of 125 post-mortems at the Foundling Hospital, New York, the bronchial glands being tubercular in every case.

The manner of such infection can be easily explained by the habit of expectorating in public places. Nuttall's (*Bulletin John Hopkins Hospital*, May, 1891,) experiments in counting the number of bacilli thrown off in the sputum from a patient with moderately advanced tubercular disease give us the astonishing figures of from one and one-half to four and one-third billion every twenty-four hours. The dangerous character of such expectoration, taken in consideration with the frequency and freedom of the habit of expectorating in this country, is perhaps the explanation of the frequency of infection by way of the respiratory system.

The danger lies not so much in the open air as in dwellings, hotels, saloons, theatre corridors, street cars and public buildings where direct sunlight cannot penetrate. Sternberg (*Manual of Bacteriology*) says that the tubercle bacillus is especially susceptible to

ORR.—Tubercle bacilli in sputum stained with carbolic fuchsin and methylene blue. Spencer 1 12 oil immersion.

the action of direct sunlight, it being killed in from a few moments to several hours, depending upon the thickness of the layer, and by diffused light, when placed near a window, in from five to seven days, while in an ordinary dwelling room it retains its virulent power for two and one-half months, gradually losing it after that time. It would seem, then, that attention and even legislation should be brought to bear upon this subject, so that the danger may be reduced to a minimum. On March 25, 1897, the Rochester board of health had an ordinance passed prohibiting expectoration on sidewalks and in public places and although the ordinance is so framed that violaters cannot be prosecuted considerable good is accomplished indirectly, as the street car company had large cards printed with the ordinance in full and posted them in their cars.

To form an idea of how widely the tubercle bacilli are spread in sputa gathered at random, I collected forty-eight samples taken from various public halls and corridor floors of this city. Three of these samples upon examination were found to contain the bacilli in such number as to make diagnosis positive.

In 1895, Dr. W. G. Bissell, city bacteriologist, made a collection of fifty-six samples from the street cars of this city, finding four of that number containing tubercle bacilli, an average of seven and fifteen one-hundredths per cent., while in my lot the percentage was five and fifteen one-hundredths. Estimating the average of the two lots it would seem that out of every one hundred expectorations there are six which contain the bacilli.

The ways in which this infected sputum may be carried about are various. It may become dry and ground into a dust and so float in the atmosphere. Women may carry it home on the edges of their dresses and so scatter it on the floors, or it may be brushed off with the dirt in the process of cleaning and so infect the air. Shoes, clothing, food, drink, all may become infected by particles of dust on which the bacteria float as long as the present habit of expectoration exists.

The quarantine department of the marine hospital service requires an army of men and vast expenditures of money to shut out infectious diseases. Yet tuberculosis is found to such an extent that of the total mortality it claims 15 per cent. If our government should start a crusade against this disease, simply giving attention to the unhygienic practice of expectoration at random in public places and walks and enforcing the rulings with the same energy that is shown by the marine hospital service, it is probable that the mortality of tuberculosis would be reduced from the present percentage to a much lower figure.

The manifestation of this disease elsewhere than in the lungs are probably in many, if not a large majority of cases, caused by material infected by the discharges (sputa) of the pulmonary form. Attacks on the latter form are indirect attacks on the former.

It is to be hoped that the departments of health in our cities will soon be vested with sufficient power to take up this question and enforce some sort of control over the habit of expectorating in public buildings, conveyances or on sidewalks.

I am indebted to Dr. Herbert U. Williams, director of the pathological laboratory for having assisted and verified this work.

## Figures and Tables

**Figure f1:**
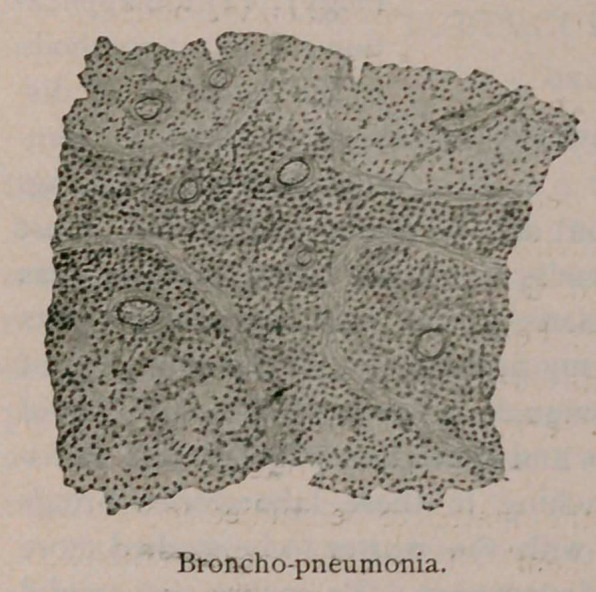


**Figure f2:**
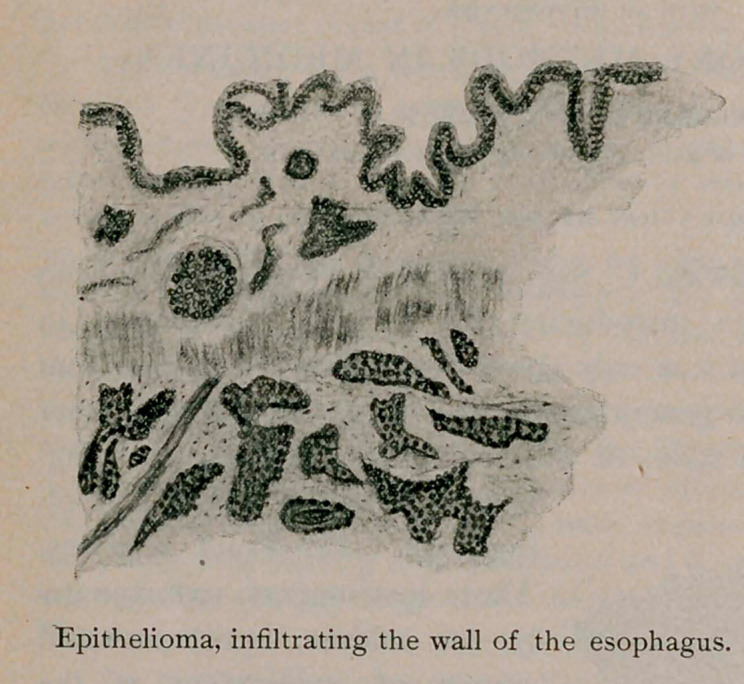


**Figure f3:**